# Genetic/Epigenetic Modulation, Ocular Diseases, and Therapeutic Prospective

**DOI:** 10.1155/2013/980608

**Published:** 2013-12-08

**Authors:** Jingsheng Tuo, Lai Wei, Nan Hu

**Affiliations:** ^1^Laboratory of Immunology, National Eye Institute, National Institutes of Health, Bethesda, MD 20892-1857, USA; ^2^State Key Laboratory of Ophthalmology, Zhongshan Ophthalmic Center, Sun Yat-sen University, Guangdong, China; ^3^Eye Institute, Affiliated Hospital of Nantong University, Nantong, China

Complex eye diseases often have significant genetic components. Previous work exploring the genetic contributions of ocular diseases has implicated numerous genomic regions and a variety of candidate genes as modulators of the disease susceptibility, including cataract, age-related macular degeneration (AMD), diabetic retinopathy (DR), glaucoma, high myopia, and others. With the advance of techniques both on genotyping and phenotyping, additional genes with a role in complex eye disease are waiting to be discovered. In contrast, it is apparent that a significant portion of the heritability of ocular disease cannot be explained through the alteration of DNA sequences. The field of epigenetics pursues the changes in gene expression or cellular phenotypes caused by mechanisms other than changes in the underlying DNA sequence. In general, epigenetic changes pertain to DNA methylation and histone modification. Aberrant epigenetic changes are associated with genomic instability and have been implicated in various human diseases. Recent advances in high-throughput platforms can generate voluminous data, which requires desperately the tools of system biology to effectively elucidate the true pictures underlying them. Knowledge and understanding of these genetic components and pathways have led to the development of promising therapies including small inference RNA (siRNA).

This special issue contains 5 articles, the contents of which are summarized as follows.

In the original paper “*An extensive replication study on three new susceptibility loci of primary angle closure glaucoma in Han Chinese: Jiangsu Eye Study*” by A. Shi et al., the authors tried to replicate recent findings of three new susceptibility loci for primary angle closure glaucoma (PACG) reported by a genome-wide association study. For a long time, the genetic study on glaucoma has been focused on primary angle open glaucoma. Instead of using clinical diagnosis of PACG as the phenotype to study, the authors chose a preclinical condition, primary angle closure (PAC), and same anatomical features of eyes to investigate. This community-based study did not find any significant association between the defined phenotypes and the single nucleotide polymorphisms in PLEKHA7, COL11A1, and PCMTD1-ST18.

In the review paper “*Vascular adhesion protein 1 in the eye*” by W. Luo et al., the authors gave an overview on the new research progresses of VAP-1 in the ocular diseases including uveitis, AMD, DR, and ocular tumor. Based on the properties and results obtained so far from preclinical and clinical studies VAP-1 may provide a novel research direction or a potent therapeutic strategy for ophthalmological diseases.

In the original paper “*RNA interference targeting connective tissue growth factor inhibits the transforming growth factor-*β*2 induced proliferation in human Tenon capsule fibroblasts*” by J. Jing et al., the authors showed that siRNA could efficiently prevent TGF-*β*2 induced proliferation of human Tenon capsule fibroblast, through targeting CTGF gene expression. Therefore, a siRNA based therapeutic approach was proposed for eliminating filtration bleb scarring after glaucoma filtration surgery.

In the original paper “*RNA interference targeting snail inhibits the transforming growth factor *β*2-induced epithelial-mesenchymal transition in human lens epithelial cells*” by P. Li et al., the authors tested the concept to use Snail targeting siRNA to block TGF *β*2-induced proliferation in human lens epithelial cells. The results show that epithelial-mesenchymal transition was inhibited by Snail targeting siRNA in the model system that the article described, accompanied by the suppression on snail expression. The finding is informative for the design of the preventive strategy on posterior capsule opacification after cataract surgery.

In the original paper “*Systems biology profiling of AMD on the basis of gene expression*” by M. S. Abu-Asab et al., a systems biology analytical paradigm called parsimony phylogenetics was used to reveal the various transcriptomic profiles of AMD's subtypes. Genetic pathways underlying the initiation and progression of AMD and the correlations of AMD's genotypes, phenotypes, and disease spectrum were investigated.

On the whole, the papers contained in this special issue covered the most active fields of genetic studies on complex eye diseases.

*Jingsheng  Tuo* *Jingsheng  Tuo* *Lai  Wei* *Lai  Wei* *Nan  Hu* *Nan  Hu*

